# Caffeine intake from different dietary sources and its association with sleep quality in employed adults

**DOI:** 10.1186/s40795-026-01312-5

**Published:** 2026-04-24

**Authors:** Kadriye Toprak, Zeyneb Yildirim, Feride Ayyildiz

**Affiliations:** 1 Department of Nutrition and Dietetics, School of Health Sciences, Ankara Medipol University, Ankara, Turkey; 2 Department of Nutrition and Dietetics, Institute of Health Sciences, Ankara Medipol University, Ankara, Turkey; 3 Department of Nutrition and Dietetics, School of Health Sciences, Gazi University, Ankara, Turkey

**Keywords:** Caffeine, Caffeine intake, Pittsburgh Sleep Quality Index, PSQI, Sleep quality

## Abstract

**Background:**

This study aimed to determine the frequency and amount of consumption of caffeine-containing foods among adults in active employment and to examine the relationship between total daily caffeine intake and sleep quality.

**Methods:**

This cross-sectional study included 428 working adults aged 19–50 years. Caffeine intake was assessed using a quantitative consumption frequency form created by the researchers, while sleep quality was assessed using the Pittsburgh Sleep Quality Index (PSQI). Statistical analyses involved appropriate parametric and nonparametric tests, as well as correlation and multiple linear regression models.

**Results:**

The participants’ average daily caffeine intake was 262.5 [144.0-411.0] mg. The primary sources of caffeine were black coffee (filter/Americano), black tea, and Turkish coffee. Participants with poor sleep quality (71.7%) consumed significantly more caffeine daily than those with good sleep quality. A positive and significant correlation was observed between caffeine intake and PSQI scores. Regression analysis indicated that caffeine consumption, was a significant independent predictor of poor sleep quality (β = 0.147, *p* = 0.003), particularly affecting sleep duration and disturbances.

**Conclusion:**

These results suggest that caffeine intake is a significant lifestyle factor independently associated with sleep quality, and a comprehensive evaluation of consumption sources could benefit public health. Future research should also focus on the timing of caffeine consumption and incorporate objective sleep measures.

**Graphical Abstract:**

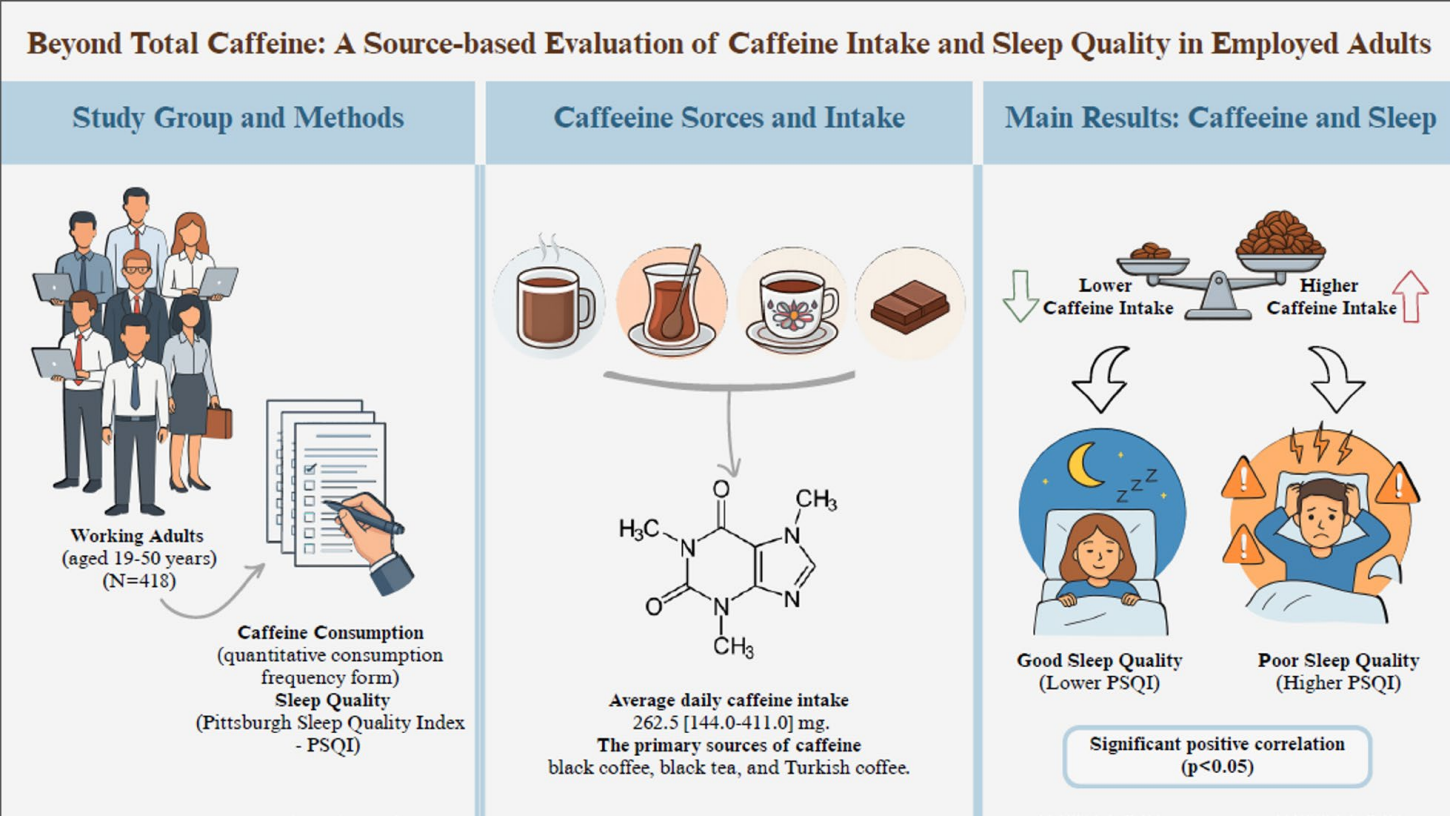

## Introduction

Diet plays a critical role in sleep quality, influencing both duration and efficiency through the timing, composition, and amount of food intake. Among dietary factors, caffeine is one of the most widely consumed psychoactive substances globally, found naturally or added to products such as coffee, tea, energy drinks, and carbonated beverages [1–3]. Due to its stimulant effects, it is frequently selected to boost alertness, reduce fatigue, and improve cognitive performance [4]. The European Food Safety Authority (EFSA) states that a daily caffeine intake of up to 400 mg is considered safe for healthy adults [5]. Nevertheless, individual consumption patterns and habits vary widely based on cultural and personal preferences.

In many populations, caffeine sources constitute a significant portion of daily fluid intake, and consumption patterns are generally shaped by sociodemographic factors and availability [6–8]. While coffee is the dominant source in Western societies, tea makes the largest contribution to daily caffeine intake in other regions, including Türkiye [9–11]. Understanding these population-level patterns is crucial for assessing the broader health impacts of caffeine, as the primary source of intake can influence total daily dose and timing of consumption.

Despite its cognitive benefits, caffeine can significantly interfere with sleep architecture. The impact of caffeine on sleep results from its antagonistic action, which blocks adenosine receptors. While excessive caffeine intake can enhance alertness, it can also negatively impact sleep duration, efficiency, and quality. Several epidemiological and experimental studies also suggest that this condition is associated with decreased sleep quality [3, 12–14]. Conversely, limiting caffeine intake in the afternoon and especially before bedtime has been shown to improve sleep quality [15]. Sleep quality encompasses multiple dimensions, including sleep duration, latency, efficiency, and fragmentation, and it has a direct impact on health at both individual and societal levels. Poor or insufficient sleep can lead to adverse consequences, including fatigue, reduced attention, functional problems, and an increased incidence of workplace accidents [16, 17]. Therefore, understanding the link between sleep and caffeine intake is crucial.

Although previous research has explored the connection between caffeine and sleep, these studies mainly focused on overall caffeine consumption while overlooking detailed analyses of specific intake sources, frequency, and their interaction with sociodemographic factors [18–20]. Additionally, a significant portion of existing literature relies on student populations, leaving a gap in data regarding actively employed adults [19, 20]. To the best of our knowledge, no comprehensive study has yet been conducted in Türkiye to simultaneously investigate diverse caffeine sources, consumption levels, and their association with sleep quality within this specific demographic. Therefore, this study aims to evaluate the frequency and quantity of caffeine intake from various dietary sources, among actively employed adults, and to investigate the relationships between these variables and sleep quality, while accounting for various sociodemographic characteristics.

## Methods

### Participants

This cross-sectional study was conducted between December 2023 and June 2024 among individuals aged 19 to 50 working in public institutions in Ankara. The study’s purpose was explained to all participants, and they were asked to participate voluntarily and sign an informed consent form. The minimum sample size was determined based on a power analysis referencing the study by Watson et al. [3], and the final study population consisted of 428 volunteers aged 19 to 50 with cognitive competence. Illiterate individuals, did not volunteer, or had any hepatic, pulmonary, renal, metabolic, psychiatric, or oncological conditions affecting nutritional status were excluded. Ethical approval for the study was obtained from the Ankara Medipol University Non-Interventional Clinical Research Ethics Committee on December 11, 2023, with decision number E-81477236-604.01.01-9185. All procedures were conducted in accordance with the principles outlined in the Declaration of Helsinki.

### Data collection instruments

During data collection, face-to-face interviews were conducted with participants, and a structured questionnaire was used to collect sociodemographic characteristics, anthropometric measurements, a food consumption frequency form developed by the researchers to assess caffeine intake, and the Pittsburgh Sleep Quality Index (PSQI).

### Assessment of caffeine intake

In the absence of a validated and reliable Turkish tool for assessing caffeine intake, a specific quantitative consumption form was developed to include sources such as coffee varieties, tea, energy drinks, soft drinks, and foods such as chocolate and cake. The final questionnaire consisted of 30 items categorized into coffee types (e.g., Turkish coffee, espresso, filter coffee, instant coffee), teas (black, green, and iced tea), caffeinated dairy products, various chocolate types, chocolate-based snacks (biscuits, cakes, and puddings), and sugar-sweetened beverages. Content validity was supported by consulting five expert dietitians. Based on participants’ self-reported consumption frequency (using nine categories ranging from ‘never’ to ‘more than 6 times per day’) and portion sizes, the total daily caffeine intake (in milligrams per day) was calculated. The caffeine content of foods was estimated using data from the USDA FoodData Central database, and individual daily caffeine intake was determined based on these estimates [21]. Additionally, to evaluate the population-level impact, the relative contribution of each caffeine source to the total daily intake was calculated by dividing the mean intake of that specific source in the total sample by the overall mean daily caffeine intake of the entire study population, expressed as a percentage.

### Pittsburgh Sleep Quality Index (PSQI)

Sleep quality in the study was evaluated using the PSQI, developed by Buysse et al. [22] and validated in Turkish by Agargun et al. [23]. The index comprises 24 items: 19 self-reported and 5 partner-reported. The total score ranges from 0 to 21, where scores of 5 and above indicate poor sleep quality and potential sleep disorders. The self-report questions consist of seven components: subjective sleep quality, sleep latency (time to fall asleep), sleep duration, habitual sleep efficiency, sleep disturbances, sleep medication use, and daytime dysfunction. Each component is scored from 0 to 3, and the sum of the seven components creates a total PSQI score ranging from 0 to 21. A total score of 5 or higher indicates ‘poor’ sleep quality and potential sleep disturbances, with higher scores reflecting greater severity of sleep quality impairment [22].

### Anthropometric data

Participants’ height and body weight were self-reported and recorded. In this study, Body Mass Index (BMI) was used to evaluate individuals’ body weight status. BMI was calculated by dividing body weight (kg) by the square of height (m²). According to the World Health Organization classification, a BMI of < 18.5 kg/m² is classified as ‘underweight’, 18.5–24.9 kg/m² as ‘normal’, 25.0–29.9 kg/m² as ‘overweight’, and ≥ 30.0 kg/m² as ‘obese’ [24].

### Physical activity status

The physical activity status of the participants was evaluated based on their self-reported response (Yes/No) to a single question regarding their engagement in regular physical activity outside of working hours.

### Statistical analysis

Data collected during the study were analyzed using IBM SPSS Statistics 26.0 [25]. The Kolmogorov–Smirnov test was used to assess the normality of the variables. Based on the distribution of the data, descriptive statistics for continuous variables were presented as median and interquartile range (Q1–Q3), while categorical variables were presented as counts (n) and percentages (%). To examine differences between two independent groups (e.g., good vs. poor sleep quality), the Mann–Whitney U test was employed. For comparisons involving more than two groups the Kruskal–Wallis test was used. The relationship between continuous variables was evaluated using Spearman correlation analysis. Finally, to evaluate the predictive value of daily caffeine intake on sleep quality, multiple linear regression analyses were performed. In these models, the total PSQI score and its seven component scores were treated as continuous outcome variables. For each outcome, a Crude Model (unadjusted) and an Adjusted Model-controlling for age, gender, educational status, exercise status, and presence of chronic disease-were computed. A significance level of *p* < 0.05 was considered significant for all analyses.

## Results

A total of 428 participants were included in the study. The median age was 27.0 [23.0–36.0] years, with 76.4% being female and 23.6% male. Most participants held college or graduate degrees, and the median BMI was 23.4 [21.5–26.3] kg/m². The average daily caffeine intake among participants was 262.5 [144.0-411.0] mg. A substantial majority of the individuals (71.7%) was classified as having poor sleep quality according to the global PSQI score (Table 1).

**Table 1:** General characteristics of the participants according to sleep quality (*n* = 428)

Variables	Sleep Quality			
	Good (*n* = 121)	Poor (*n* = 307)	Total (*n* = 428)	*p*
Gender (%)				
Male	17 (86.0)	84 (27.4)	101 (23.6)	**0.003**
Female	104 (14.0)	223 (72.6)	327 (76.4)	
Age (Median [Q_1_-Q_3_])	29.0 [25.0–42.0]	26.0 [22.0–35.0]	27.0 [23.0–36.0]	**0.003**
Education Level (%)				
Primary education	29 (24.0)	45 (14.7)	74 (17.3)	**0.042**
High school	28 (23.1)	96 (31.3)	124 (29.0)	
Undergraduate-Graduate	64 (52.9)	166 (54.1)	230 (53.7)	
BMI (Median [Q_1_-Q_3_])	23.4 [21.9–25.9]	23.4 [21.3–26.6]	23.4 [21.5–26.3]	0.998
Underweight (< 18.5 kg/m²)	6 (5.0)	9 (2.9)	15 (3.5)	0.171
Normal weight (18.5–24.9 kg/m²)	80 (66.1)	193 (62.9)	273 (63.8)	
Overweight (25.0–29.9 kg/m²)	18 (14.9)	72 (23.5)	90 (21.0)	
Obese (≥ 30.0 kg/m²)	17 (14.0)	33 (10.7)	50 (11.7)	
Physical Activity Status (%)				
Yes	121 (100.0)	289 (94.1)	410 (95.8)	**0.007**
No	0 (0.0)	18 (5.9)	18 (4.2)	
Presence of any disease (%)				
Yes	60 (49.6)	213 (69.4)	155 (36.2)	**< 0.001**
No	61 (50.4)	94 (30.6)	273 (63.8)	
Caffeine Intake (mg/day)	176.1 [136.5-292.5]	292.8 [145.4–430.0]	262.5 [144.0-411.0]	**< 0.001**
PSQI Total	3.0 [3.0–4.0]	8.0 [6.0–10.0]	6.0 [4.0–9.0]	**< 0.001**
Subjective sleep quality	1.0 [0.0–1.0]	2.0 [0.0–3.0]	2.0 [1.0–3.0]	**< 0.001**
Sleep latency	1.0 [0.0–1.0]	2.0 [1.0–3.0]	1.0 [0.0–2.0]	**< 0.001**
Sleep duration	0.0 [0.0–1.0]	1.0 [0.0–2.0]	1.0 [0.0–1.0]	**< 0.001**
Habitual sleep efficiency	0.0 [0.0–1.0]	1.0 [0.0–1.0]	1.0 [0.0–1.0]	**< 0.001**
Sleep disturbances	1.0 [0.0–1.0]	2.0 [1.0–3.0]	1.0 [1.0–3.0]	**< 0.001**
Use of sleeping medication	0.0 [0.0–0.0]	0.0 [0.0–0.0]	0.0 [0.0–0.0]	**-**
Daytime dysfunction	0.0 [0.0–1.0]	1.0 [0.0–1.0]	1.0 [0.0–1.0]	**< 0.001**

Pearson Chi kare. *BMI* Body Mass Index, Bold values indicate statistical significance

Table 2 presents the participants’ caffeine intake based on various variables. Those who engaged in physical activity were found to have lower caffeine intake (*p* < 0.05).

**Table 2:** Caffeine intake levels according to selected variables (*n* = 428)

Variables	Caffeine Intake Median [Q_1_-Q_3_]	*p*
Gender		
Male	286.1 [120.5-401.7]	0.818
Female	251.5 [144.5-419.6]	
Education Level		
Primary education	245.6 [141.3-346.9]	0.085
High school	303.4 [145.4-469.5]	
Undergraduate-Graduate	245.4 [144.0-392.8]	
BMI Categories		
Underweight (< 18.5 kg/m²)	198.3 [53.0-653.8]	0.166
Normal weight (18.5–24.9 kg/m²)	239.0 [134.4-453.5]	
Overweight (25.0–29.9 kg/m²)	303.1 [171.0-430.8]	
Obese (≥ 30.0 kg/m²)	260.3 [155.4-334.5]	
Physical Activity Status		
Yes	256.0 [141.0-409.5]	**0.021**
No	346.8 [263.4-584.5]	
Presence of any disease		
Yes	279.5 [144.0-419.5]	0.685
No	248.1 [141.4-409.5]	
PSQI		
Good sleep quality	176.1 [136.5-292.5]	**< 0.001**
Poor sleep quality	292.8 [145.4–430.0]	

Mann-Whitney U, Kruskal Wallis, *BMI* Body mass index, *PSQI* Pittsburgh Sleep Quality Index, Bold values indicate statistical significance

The frequencies of participants’ consumption of caffeine-containing foods and drinks are shown in Table 3. While 19.6% of participants reported drinking more than 6 cups of black tea daily, 9.6% stated that they never consumed black tea at all. When examining the frequency of coffee consumption, Turkish coffee stood out, with approximately two-thirds of participants (61.7%) reporting that they drank it daily or weekly.

**Table 3:** Consumption frequency of caffeine-containing foods and beverages (%)

	> 6 times/day	4–6 times/day	2–3 times/day	1 time/day	5–6 times/week	2–4 times/week	1 times/week	1–3 times/month	Never
Espresso	0 (0.0)	0 (0.0)	8 (1.9)	0 (0.0)	8 (1.9)	9 (2.1)	17 (4.0)	31 (7.2)	355 (82.9)
Black coffee (Filter/Americano)	0 (0.0)	4 (0.9)	53 (12.4)	41 (9.6)	41 (9.6)	26 (6.1)	26 (6.1)	60 (14.0)	177 (41.4)
Instant coffee	0 (0.0)	0 (0.0)	23 (5.4)	5 (1.2)	16 (3.7)	11 (2.6)	14 (3.3)	39 (9.1)	320 (74.8)
Turkish coffee	0 (0.0)	7 (1.6)	51 (11.9)	71 (16.6)	13 (3.0)	70 (16.4)	52 (12.1)	71 (16.6)	93 (21.7)
Caffe Latte	0 (0.0)	0 (0.0)	15 (3.5)	38 (8.9)	8 (1.9)	39 (9.1)	40 (9.3)	53 (12.4)	235 (54.9)
Coffee with milk	0 (0.0)	0 (0.0)	6 (1.4)	0 (0.0)	0 (0.0)	8 (1.9)	12 (2.8)	52 (12.1)	350 (81.8)
Chocolate milk	0 (0.0)	0 (0.0)	0 (0.0)	0 (0.0)	0 (0.0)	0 (0.0)	8 (1.9)	43 (10.0)	377 (88.1)
Black tea	84 (19.6)	100 (23.4)	129 (30.1)	38 (8.9)	11 (2.6)	19 (4.4)	3 (0.7)	3 (0.7)	41 (9.6)
Green tea	0 (0.0)	4 (0.9)	15 (3.5)	22 (5.1)	7 (1.6)	37 (8.6)	34 (7.9)	47 (11.0)	262 (61.2)
Iced tea	0 (0.0)	0 (0.0)	4 (0.9)	0 (0.0)	10 (2.3)	19 (4.4)	24 (5.6)	34 (7.9)	337 (78.7)
Cola drinks	0 (0.0)	0 (0.0)	19 (4.4)	0 (0.0)	14 (3.3)	46 (10.7)	35 (8.2)	54 (12.6)	260 (60.7)
Energy drinks	0 (0.0)	0 (0.0)	6 (1.4)	0 (0.0)	0 (0.0)	10 (2.3)	0 (0.0)	41 (9.6)	371 (86.7)
Dark chocolate	0 (0.0)	0 (0.0)	13 (3.0)	27 (6.3)	10 (2.3)	52 (12.1)	51 (11.9)	105 (24.5)	170 (39.7)
Milk chocolate	0 (0.0)	0 (0.0)	8 (1.9)	8 (1.9)	13 (3.0)	41 (9.6)	60 (14.0)	108 (25.2)	190 (44.4)
White chocolate	0 (0.0)	0 (0.0)	3 (0.7)	0 (0.0)	0 (0.0)	4 (0.9)	0 (0.0)	19 (4.4)	402 (93.9)
Chocolate bar	6 (1.4)	0 (0.0)	23 (5.4)	53 (12.4)	23 (5.4)	89 (20.8)	41 (9.6)	52 (12.1)	141 (32.9)
Coffee ice cream	0 (0.0)	0 (0.0)	0 (0.0)	4 (0.9)	0 (0.0)	0 (0.0)	0 (0.0)	35 (8.2)	389 (90.9)
Chocolate ice cream	0 (0.0)	0 (0.0)	0 (0.0)	10 (2.3)	0 (0.0)	14 (3.3)	11 (2.6)	86 (20.1)	307 (71.7)
Chocolate biscuits	6 (1.4)	0 (0.0)	9 (2.1)	7 (1.6)	25 (5.8)	47 (11.0)	67 (15.7)	102 (23.8)	165 (38.6)
Chocolate and coffee biscuits	0 (0.0)	0 (0.0)	3 (0.7)	0 (0.0)	8 (1.9)	10 (2.3)	11 (2.6)	62 (14.5)	334 (78.0)
Chocolate sauce	0 (0.0)	0 (0.0)	3 (0.7)	0 (0.0)	0 (0.0)	0 (0.0)	7 (1.6)	100 (23.4)	318 (74.3)

Table 4 presents the relative contributions of various food and beverage sources to the total daily caffeine intake, along with consumer frequencies and mean intake amounts among consumers. Black coffee (Filter/Americano) was the primary contributor to the total caffeine intake (23.9%), followed by black tea (21.8%) and Turkish coffee (12.5%). Although the mean intake among espresso consumers was the highest (185.1 ± 291.6 mg/day), its overall contribution to the total sample’s intake was limited to 10.2% due to its lower consumption frequency (17.1%). The contributions of other sources to total caffeine intake were 4% or less of the total.

**Table 4:** Distribution and frequency of food and beverage sources contributing to daily total caffeine intake (*n* = 428)

	Consumers *n* (%)*	Intake among consumers (mg/day) (X̄±SD)**	Intake in total sample (mg/day) (X̄ ±SD)***	Contribution to total intake (%)
Black coffee (Filter/Americano)	251 (58.6)	127.1 ± 133.7	74.5 ± 119.9	23.9
Black tea	387 (90.4)	75.0 ± 40.6	67.8 ± 44.5	21.8
Turkish coffee	335 (78.3)	49.6 ± 58.7	38.8 ± 55.8	12.5
Caffe latte	193 (45.1)	70.5 ± 83.7	31.8 ± 66.2	10.2
Espresso	73 (17.1)	185.1 ± 291.6	31.6 ± 138.5	10.2
Instant coffe	108 (25.2)	48.6 ± 58.4	12.3 ± 36.1	4.0
Dark chocolate	258 (60.3)	20.2 ± 28.5	12.2 ± 24.2	3.9
Coffee with milk	78 (18.2)	48.1 ± 102.2	8.8 ± 47.2	2.8
Cola drinks	168 (39.3)	16.1 ± 23.0	6.3 ± 16.4	2.0
Energy drinks	57 (13.3)	38.9 ± 74.3	5.2 ± 30.0	1.7
Green tea	166 (38.8)	9.9 ± 14.6	3.8 ± 10.3	1.2
Chocolate and coffee biscuits	94 (22.0)	12.3 ± 22.3	2.7 ± 11.6	0.9
Chocolate biscuits	263 (61.4)	4.6 ± 9.7	2.9 ± 8.0	0.9
Iced tea	91 (21.3)	9.1 ± 13.5	1.9 ± 7.2	0.6
Milk chocolate	238 (55.6)	3.0 ± 4.8	1.7 ± 3.9	0.5
Coffee ice cream	39 (9.1)	18.1 ± 28.0	1.7 ± 9.9	0.5
Chocolate bar	287 (67.1)	2.2 ± 3.0	1.5 ± 2.7	0.5
Chocolate ice cream	121 (28.3)	1.0 ± 1.3	0.3 ± 0.8	0.1
Chocolate sauce	110 (25.7)	0.6 ± 0.9	0.2 ± 0.5	0.1
Chocolate milk	51 (11.9)	0.5 ± 0.2	0.1 ± 0.2	0.0
White chocolate	26 (6.1)	2.1 ± 2.8	0.1 ± 0.8	0.0

*Any consumption (mg/day > 0). **Mean intake among consumers only. ***Mean intake in the total sample. Contribution to total intake (%): based on per-capita means. Calculated as (mean intake of the specific source in the total sample / overall mean daily caffeine intake of the total sample) x 100

Table 5 presents multiple linear regression analyses of the dose-response relationship between daily caffeine intake and components of sleep quality. When potential confounding factors such as age, gender, educational status, exercise status, and chronic disease presence were included in the model and adjusted for, increased caffeine consumption was significantly associated with poorer overall sleep quality (*p* = 0.003). When examining the PSQI components, high caffeine intake was found to have a statistically significant confounding effect specifically on ‘sleep duration’ (*p* = 0.029) and ‘sleep disturbances’ (p = 0.002).

**Table 5:** Multiple linear regression analyses evaluating the predictive value of daily caffeine intake on global and components of sleep quality (*n* = 428)

PSQI and Components	Crude Model^a^				Adjusted Model^b^			
	B	SE	β	*p*	B	SE	β	*p*
PSQI Score (Total)	0.0020	0.0007	0.147	0.002	0.0020	0.0007	0.147	0.003
Subjective sleep quality	0.0003	0.0002	0.066	0.170	0.0003	0.0002	0.069	0.161
Sleep latency	0.0004	0.0002	0.088	0.068	0.0003	0.0002	0.075	0.127
Sleep duration	0.0004	0.0002	0.101	0.037	0.0004	0.0002	0.107	0.029
Habitual sleep efficiency	0.0003	0.0002	0.085	0.079	0.0003	0.0002	0.081	0.101
Sleep disturbances	0.0007	0.0002	0.151	0.002	0.0007	0.0002	0.148	0.002
Use of sleeping medication^c^	-	-	-	-	-	-	-	-
Daytime dysfunction	0.0001	0.0002	0.014	0.766	0.0001	0.0002	0.027	0.586

*PSQI* Pittsburg Sleep Quality Index, *B* Unstandardized regression coefficient, *SE* Standard Error, *β* Standardized regression coefficient

^a^Crude model: Unadjusted model with caffeine intake (mg/day) as the sole independent predictor

^b^Adjusted model: Adjusted for age (continuous), gender, educational status, exercise status, and presence of chronic disease

^c^Linear regression analysis could not be computed for ‘Use of sleeping medication’ due to zero variance, as all participants reported no use of sleep medication

## Discussion

This study aimed to evaluate the frequency and quantity of caffeine consumption from various dietary sources among actively employed adults, and to examine the relationship between total caffeine intake and sleep quality. Our analysis revealed a remarkably high prevalence of poor sleep quality (71.7%) within the study population. While caffeine consumption was highly prevalent among participants, black tea (90.4%) and Turkish coffee (78.3%) were the most frequent sources, though black coffee (filter/Americano) was the leading contributor to total daily intake (23.9%). Regression analyses showed that increased daily caffeine intake was significantly associated with poorer overall sleep quality (*p* = 0.003), specifically impacting the ‘sleep duration’ (*p* = 0.029) and ‘sleep disturbances’ (p = 0.002) components. Distinct from previous research limited to basic associations, this study distinguishes itself by examining the relationship between caffeine intake and sleep quality within a multidimensional framework. While studies focusing on total caffeine amount predominate in the literature [18–20], this study evaluates caffeine intake in detail, incorporating consumption frequency and sociodemographic variables. In addition, examining actively employed adults provided valuable insights into the interaction between caffeine intake and the dynamics of daily professional life. In this respect, the study diverges from the prevailing research on student populations and makes a significant contribution by evaluating caffeine intake in relation to various occupational and lifestyle factors. The findings indicate that caffeine intake is widespread and primarily comes from hot beverages, such as black coffee (filter/Americano), black tea, and Turkish coffee. Furthermore, increased daily caffeine intake was found to be a significant predictor of poorer sleep quality. These findings align with the current literature, reinforcing the association between excessive caffeine consumption and reduced sleep quality [1, 3, 12, 19, 26].

The study found that over 90% of participants consumed at least one caffeinated product, with black tea (90.4%) and Turkish coffee (78.3%) being the most commonly consumed beverages. These findings are consistent with studies indicating that caffeine intake generally relies on traditional sources such as tea and coffee [16, 27]. Black tea and Turkish coffee are traditional in our country and among the most frequently consumed beverages [28, 29]. Although black tea was identified as the most frequently consumed beverage, black coffee (filter/Americano) account for the primary contributor to total caffeine intake, despite its lower consumption frequency than tea. This finding aligns with previous studies that have identified coffee as the primary source of dietary caffeine [12, 30, 31]. These findings demonstrate that a product’s contribution to total caffeine intake is a function of consumption frequency, portion size, and caffeine concentration, rather than frequency alone. Consequently, this research makes a significant contribution to the literature by providing a detailed evaluation of specific dietary sources in conjunction with total caffeine volume.

The high prevalence of poor sleep quality, observed in 71.7% of participants, is one of the study’s striking findings. This rate is significantly higher than those reported in general population studies in Türkiye, which typically range from 15.0% to 50% [32–34]. This difference suggests that the actively employed population in our sample may face unique challenges that significantly impair sleep hygiene compared to the general public. Reasons for this high prevalence include intense work-related stressors, demanding professional schedules, and the dynamics of working life that necessitate caffeine consumption to maintain alertness throughout the day [33, 35–39]. Similar high rates of sleep disturbances have been observed in other studies of high-stress occupational groups, supporting the idea that professional life significantly burdens sleep quality [35, 37].

In addition to traditional beverages, the consumption of coffee varieties such as filter coffee, Americano, and espresso has also been increasing in our country, especially among young adults [40]. In addition to traditional beverages in our country, the consumption of coffee varieties such as filter coffee, Americano, and espresso has also increased recently, especially among young adults. This increase can be attributed to the spread of coffee culture and the growing social acceptance of coffee consumption. Today, coffee is consumed not only to increase alertness but also as a part of socializing and daily routines [41]. Since the sample of this study also consisted of working adults, it suggests that they also prefer black coffee (filter/Americano) in their daily work routines. Although black coffee (filter/Americano) ranked third in consumption frequency, it was identified as the primary contributor to total caffeine intake due to its significantly higher caffeine content compared to other beverages. Other dietary sources of caffeine include Turkish coffee (12.5%), cafe latte (10.2%), and espresso (10.2%). However, the percentages of these products in total caffeine intake are lower than those of black tea and black coffee. Although Turkish coffee contains more caffeine per serving than black tea, it was found that its contribution to total caffeine intake is less than that of black tea due to its lower consumption frequency compared to tea. This finding demonstrates that caffeine intake should be evaluated not only based on beverage type but also considering consumption frequency and portion size.

When examining the relationship between sleep quality and caffeine intake, it was observed that individuals with poor sleep quality had higher daily caffeine intake than those with good sleep quality. Furthermore, multiple linear regression analysis revealed that daily caffeine intake was a significant independent predictor of global PSQI scores, even after adjusting for potential confounders, including age, gender, educational status, and chronic disease. These results align with previous findings in the field. Watson et al. [3] demonstrated that high caffeine intake negatively affects both objective and subjective sleep indicators, and that adults with poor sleep quality tend to consume more caffeine. Another study also reported that caffeine intake above 400 mg/day is associated with poor sleep quality [19]. Crucially, our study examined the components of sleep quality and found that high caffeine intake was significantly associated with short ‘sleep duration’ and ‘sleep disturbances’. Similarly, the literature shows that caffeine consumption shortens total sleep duration and disrupts sleep continuity by increasing night-time awakenings [13, 42]. This can be explained by caffeine blocking A1 and A2A adenosine receptors. Caffeine accumulates during wakefulness, stimulating the central nervous system by blocking adenosine, which sends a “sleep pressure” signal to the body, making it difficult to transition into deep sleep stages and causing fragmented sleep [3, 12, 43, 44]. On the other hand, although the literature generally indicates that caffeine prolongs sleep latency [14, 45], this study found no significant relationship between sleep latency and caffeine intake. This may be related to the timing of caffeine consumption among the working population that constituted our sample. Even if high amounts of caffeine consumed early in the day fall below the threshold value that would prevent sleep onset by bedtime, it can continue to disrupt the architecture and quality of sleep throughout the night [13, 42, 46]. In addition, it has been reported that the partial tolerance that develops in regular consumers reduces the acute effect of caffeine on sleep onset [2, 45]. In conclusion the impact of coffee on sleep quality may depend not only on the amount of caffeine consumed but also on key variables such as the timing of caffeine intake, stress levels, and genetic factors [26, 46, 47]. All these findings highlight that caffeine intake is a significant and independent lifestyle factor associated with sleep quality in the working population, even after accounting for various sociodemographic and health-related variables.

A notable strength of the study is that caffeine intake was assessed in detail by product, not just as a total amount. Presenting percentages of daily caffeine intake from various foods and beverages provides an opportunity to identify which foods to target for public health interventions. Furthermore, focusing on an actively employed population offers valuable insights into the interaction between caffeine and sleep within the professional context. However, several limitations should be taken into consideration. First, due to the study’s cross-sectional design, causal inferences cannot be made. Second, participants were not asked about the times of day they consumed caffeine. This is a notable limitation, as the timing of intake is known to influence the effect of caffeine on sleep [26]. Thirdly, although multiple established confounders were adjusted for, total daily energy intake and physical activity levels, particularly physical activity performed close to bedtime, were not comprehensively assessed. These factors may independently influence sleep quality and could have resulted in residual confounding. Furthermore, physical activity was evaluated using a single-item, self-reported question rather than a validated instrument. In this desk-based population, the high prevalence of physical activity (95.8%) may be due to participants’ tendency to overreport themselves as active. The other important limitation of this study is that caffeine intake was assessed using a researcher-developed questionnaire, as no validated Turkish tool covering all relevant caffeine sources was available at the time of the study. While content validity was established through consultation with five expert dietitians, the questionnaire has not yet undergone full psychometric validation, including test-retest reliability or validity against objective gold-standard methods (e.g., biomarkers). Future studies should aim to evaluate the psychometric properties of such tools to further strengthen the assessment of caffeine consumption. Finally, obtaining the data through self-reporting may have led to recall bias. Furthermore, the fact that anthropometric measurements were also based on self-reporting may limit the accuracy of the data.

## Conclusion

In conclusion, this study offers a current and comprehensive overview of caffeine consumption frequency and quantity within the population, while also illustrating the relationship between total caffeine intake and poorer sleep quality. These findings may inform awareness campaigns aimed at maintaining sleep hygiene, particularly in populations where high caffeine intake is prevalent. Future research should explore this relationship in greater depth by incorporating caffeine intake timing and objective sleep measures. Furthermore, future longitudinal studies are warranted to elucidate the temporal and causal relationships between daily caffeine intake patterns and sleep quality outcomes.

## Data Availability

The data supporting the findings of this study are available from the corresponding authors upon request.
